# Editorial

**Published:** 2017-02-27

**Authors:** Nikhil Marwah MDS

**Affiliations:** Editor-in-Chief, International Journal of Clinical Pediatric Dentistry, Associate Editor, JMGUMST, Professor and Head, Department of Pediatric Dentistry, Mahatma Gandhi Dental College, MGUMST, Jaipur, Rajasthan, India

***A step in the correct direction.............Second prize in journal category by***

***‘The Federation of Indian Publishers’***

A very happy new year to all and hope this year ushers in the best health and wishes for all. It gives me immense pleasure to announce that our journal, International Journal of Clinical Pediatric Dentistry (IJCPD) has won the second prize in Journal category printed and published in India by ’The Federation of Indian Publishers’. It is a moment of immense pride for the entire team, editorial staff, Jaypee group, editorial board, reviewers and most importantly the authors of articles who have always trusted us and shown faith in us for their scientific manuscripts. I assure you that this is just another milestone for us and the journey will continue as we have miles to go.

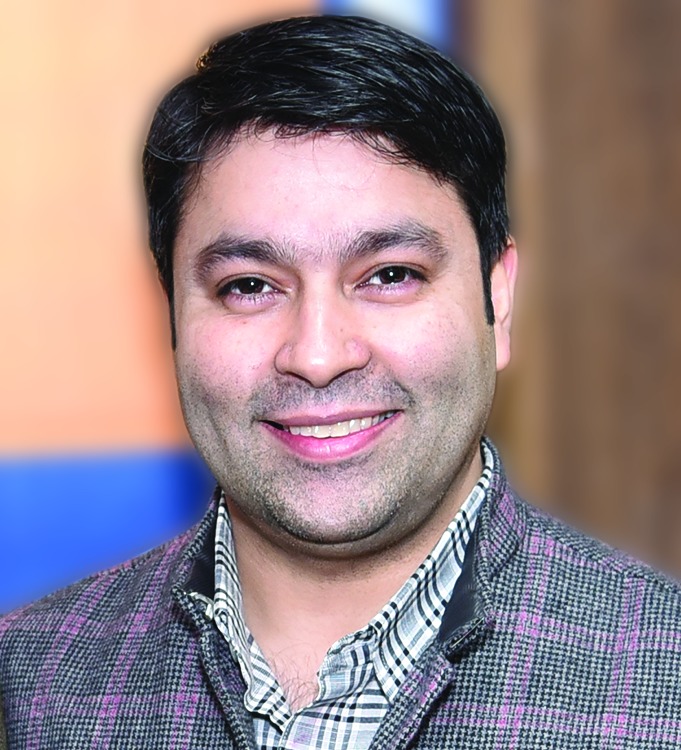



Expansion is the essence of time and we at IJCPD believe that it is always more advisable to have a large number of contributors as it increases our scientific acumen and also the journal’s outreach among society. I whole heartedly welcome Drs Satish Vishwanathaiah and Ravi GR who have joined us as Assistant Editors. International Journal of Clinical Pediatric Dentistry is also pleased to welcome new members Drs Rupinder Bhatia, Nikhil Srivastava, Srinivas Namineni, Sukhdeep Singh, Arathi Rao, Mandeep Singh, Neeraj Gugnani, Vijayprakash Mathur, Ameet J Kurthukoti, Anshula Deshpande and Puneet Goenka to its editorial board and I am sure their valuable contribution and knowledge will help the journal to achieve its further growth.

International Journal of Clinical Pediatric Dentistry is one of the few journals in the world which publishes a preliminary list of articles on its website which are going to appear in subsequent issues (until 2019). We are also in the process of making these articles available online for the authors’ use very soon. We pridely transgress the information of having a transparent and time-bound review process so that waiting time for article decisions is not prolonged for the authors and we would improvise further in future to reduce the waiting time post-acceptance by increasing the number of articles in each issue or increase the number of issues in a year. While we work to make this journal more efficient and approachable, I, once again thank all for their valuable support and love and hope that you will continue to trust us for your scientific publications.

**Nikhil Marwah** MDS

Editor-in-Chief, International Journal of Clinical Pediatric Dentistry

Associate Editor, JMGUMST

Professor and Head, Department of Pediatric Dentistry

Mahatma Gandhi Dental College, MGUMST

Jaipur, Rajasthan, India

